# The role of peer support in recovery among clients with mental illness attending the psychiatric service in a tertiary hospital in Malaysia: a qualitative study

**DOI:** 10.1186/s12888-024-05901-1

**Published:** 2024-06-26

**Authors:** Izzul Hazwan Sulaiman, Nur Iwana Abdul Taib, Jane Tze Yn Lim, Tuti Iryani Mohd Daud, Marhani Midin

**Affiliations:** 1 Department of Psychiatry, Faculty of Medicine, Universiti Kebangsaan Malaysia, Kuala Lumpur, Malaysia; 2 Department of Psychological Medicine, Faculty of Medicine and Health Sciences, Universiti Malaysia Sarawak, Kota Samarahan, Malaysia

**Keywords:** Recovery perspective, Mental health, Peer support group

## Abstract

**Background:**

The understanding that mental health recovery is a personal and subjective experience informs mental health policies in many countries. However, most of the populations in these studies are from the West, limiting their applicability in Asia. Peer support in mental health refers to helping and mentoring people who have overcome similar obstacles. Despite being proven to be effective in promoting recovery, little is known about its use in Malaysian psychiatric patients. This study aims to explore the participants’ perspectives on their concept of recovery and how the peer support group (PSG) aid them to achieve recovery.

**Methodology:**

This study was conducted on clients with mental illness who attended the PSG provided by the Community Psychiatry and Psychosocial Intervention Unit in National University Malaysia Medical Centre. A qualitative generic inductive approach was employed in this thematic exploratory study. Purposive sampling was the method used to collect the data for this thematic exploratory study. In-depth interviews of 11 study participants were audio recorded and transcribed verbatim. Data were analyzed using Braun and Clarke (2006) descriptive thematic analysis method.

**Results:**

The findings of this study highlighted six key themes; three pertaining to the participants’ perspectives on the meaning of recovery (1. *Gaining self-reliance and social inclusion*, 2. *Personal growth and improved life circumstances in recovery*, and 3. *Symptoms improvement*) and another three pertaining to how the peer support group aids recovery (1. *Empowerment and growth through peer support*, 2. *Promoting well-being*, 3. *Social connection and support*).

**Conclusion:**

The findings of this study provide valuable insights into the perspectives of psychiatry clinic patients enrolled in PSG on the concept of recovery and the role of such groups in their recovery journey. The findings demonstrated that the PSG complemented the participants’ perspectives on recovery, reinforcing the notion that a comprehensive and person-centered approach to mental health services is essential for successful and sustained recovery outcomes.

## Background

Recovery is a contested concept within the mental health arena between and within groups of service users, carers, professionals, and policymakers [1]. There is no widely accepted definition of recovery in mental health, as recovery will give different meanings to different people experiencing it. As a concept, recovery is subject to many interpretations and is therefore appropriated in different ways for different purposes. The word ‘recovery’ is used with a range of incompatible meanings; hence without conceptual clarity, a rational debate is impossible. Therefore, it is important to understand the term conceptually because the focus on recovery is advocated as the guiding principle for mental health policy in many countries, especially English-speaking countries [2]. Two forms of ‘recovery’ have been identified, namely ‘clinical recovery’ and ‘personal recovery’, which have emerged differently from professional literature and consumer narratives, respectively [2]. In the treatment and outcome monitoring of clients with severe mental illness, clinical and personal recovery should be considered concurrently.

Clinical recovery is the traditionally used definition of recovery in mental health services which focuses on sustaining remission. This puts the recovery concept within the illness frame of understanding and implies long-term reduction or ideally resolution of symptoms, followed by improvement in functioning. The important feature in this ‘recovery’ definition is that it is invariant across individuals, making it relatively easy to operationalize the concept. The definition of personal recovery has emerged from the increasingly coherent voices of individuals who have experienced mental illnesses and used mental health services. Based on recent studies, the common emerging theme is an emphasis on understanding recovery beyond the absence of illness markers of symptoms and functional impairment [3–5]. From this perspective, the popular definition based on the widely cited definition of recovery is “*a deeply personal, unique process of changing one’s attitudes, values, feelings, goals, skills and roles. It is a way of living a satisfying, hopeful, and contributing life even with limitations caused by the illness. Recovery involves the development of new meaning and purpose in one’s life as one grows beyond the catastrophic effects of mental illness”* [6]. An understanding of recovery as a personal and subjective experience has emerged within the mental health systems, which underpins mental health policies in many countries. Studies exploring mental illness from personal approach have emphasized the need for rediscovering hope and regaining a sense of self-determination [7], interconnectedness [8], goal setting [9], empowerment [10, 11], and exploring new identities [9, 12]. However, these studies have predominantly involved Western populations, so their conclusion may not be applicable in Asia. As far as the authors know, no study was done to investigate this among the Malaysian population.

Peer support has been identified to be an important element of recovery. In the context of mental health, peer support refers to providing support, encouragement, and mentoring by individuals who have personally experienced and overcome mental health challenges [13]. A study among adolescents found that peer support was one of the most important predictors of adolescents’ subjective well-being across all eight world regions [14]. In Western countries, service delivery has evolved to a point where they employ mental health clients to deliver peer support activities. Peer support workers (PSWs) have been identified as one of ten empirically validated recovery-promoting interventions [15]. PSWs are individuals recovering from mental illnesses who identify themselves positively and strongly desire to use their lived experiences to help others with similar conditions [16, 17]. In sharing and engaging with their peers, they rely on the knowledge gained through their lived experiences of mental illnesses. Such expertise cannot be replaced by professional training. Over the past two decades, peer support has been considered a critical means of empowering service users to promote their recovery process [18, 19]. Social support works as a factor that modifies the effects of stressful life events, and having good social support was significantly associated with a lower rate of rehospitalization among clients with severe mental illness [20]. Few countries in Asia had started integrating peer support services in their recovery program across various roles and positions. In Singapore, this service involves training and certification of people with lived experience as Peer Support Specialist (PSS) to develop the peer support system within the local healthcare workforce [21]. Meanwhile in Hong Kong, the role of peer support was incorporated into their supported employment program [22]. The peer support program in China is conducted in a community rehabilitation center or community health care center focusing on various recovery-oriented skills such as daily life skills and social skills [23]. The different setting of peer support programs across these Asian countries may be due to different community needs and available resources.

In Malaysia, peer support service is still in its infancy stage. While programs supporting families have started in Malaysia, peer support program is still largely absent in the system and requires development. Empowerment of people to contribute to the delivery of services must be a main feature, and the new service system should adopt the message that “mental health is everybody’s business”. Improvement of social support should receive a priority in further service development for people with severe mental illness [24]. There are no clear guidelines for implementing peer support group (PSG) in our standard routine mental health care services in Malaysia. In Community Psychiatry and Psychosocial Intervention Unit in National University Malaysia Medical Centre, PSG service was established in August 2016. It does not follow any structured modules since, as of the time of writing, its distinctive module is still being developed. The traditional method of providing this service via formal peer-support workers (PSW) was not feasible due to the novelty of peer-support group services in Malaysia, limitations on resources, and the lack of structure and policies regarding PSG services. PSG participants physically gather once every two weeks in the Community Psychiatry and Psychosocial Intervention Unit meeting room, where a qualified mental health professional (occupational therapist) will facilitate the group. Each session usually lasted one to two hours. These two weekly meetings include peers’ sharing as well as incorporating an educational component that is chosen by the peers themselves, for example, stress management or a spiritual input from a religious authority or any other helpful resources available. The number of participants for each session varies with a range of eight to twelve persons each time. Other activities include theme-based group discussions, role-playing, outdoor exercises and programs, homework-based tasks, and leisure activities. A grand meeting is held annually, commonly with more attendees, where the proposed themes and topics for the events and activities of the following year are discussed and agreed upon. When discussing related themes and topics, peer members’ needs and progress are taken into account. Mobile technology (via the WhatsApp application) is also being used for peers’ social connection within the WhatsApp application, which included group members as well as the occupational therapist who serves as a mediator.

To our current knowledge, this would be the first study conducted among clients with mental illness in Malaysia to investigate their perspectives on the meaning of recovery and how the PSG aids them in achieving recovery. The findings of this study will also contribute to the existing body of knowledge regarding the experiences and perspectives of individuals with mental illness in Asian countries, particularly Malaysia, as there is a lack of in-depth exploration of the factors that may influence the understanding and experience of recovery in Asian cultural contexts. Understanding the positive values identified in the current module of the PSG in National University Malaysia Medical Centre would aid in developing effective strategies for introducing and implementing the PSG modules in our national mental health services.

## Methodology

This thematic exploratory study employed a qualitative generic inductive approach using Caelli’s generic principle [25]. Such a design allows for a more in-depth insight into the participants’ perspective on their concept of recovery and how a PSG helps them achieve it. The study was conducted from December 2022 until February 2023 on the current clients with mental illness who attended the PSG service provided by the Community Psychiatry and Psychosocial Intervention Unit in National University Malaysia Medical Centre.

This study employed a purposive sampling method. Purposive sampling effectively identifies and selects information-rich cases in settings with limited resources. Inclusion criteria were peers who were: (1) 18 years old or older; (2) diagnosed to have a mental illness and are under psychiatry clinic follow-up; (3) able to converse in either Malay or English language; (4) mentally capable (in accordance to Mental Health Capacity Act) to give written informed consent to participate in the study; and (5) interested in reflecting their experiences of receiving PSG service. The exclusion criteria were peers; 1) mentally incapable of providing informed consent to participate in the study.

All clients attending the PSG service who met the study’s inclusion and exclusion criteria were invited to participate in the study during their PSG sessions. The participants were explained on the study’s procedures, purposes, benefits and risks, assurance of anonymity, and their right to withdraw from the study at any point in time; before signing an informed consent form. Participants who have provided their consent were contacted individually to arrange an appointment for in-depth interview with the researchers at an agreed-upon time between both parties.

In-depth interviews (IDI) were used for primary data collection. Several factors support the use of in-depth interviews as the principal method in this study instead of focus group discussion (FGD). In-depth interviews allow for a deeper understanding of members’ experiences and opinions, providing detailed and nuanced insights into their perspective on recovery and how the PSG aids them in achieving recovery. In-depth interviews also allowed participants to reflect on their personal journeys, discuss their ideas, emotions, and impressions, and provide contextualized explanations of their experiences through open-ended questions and questioning. It also allows for clarification and questioning, allowing us to go further into participants’ comments and acquire a more comprehensive insight into their experiences. Two mental health professional doctors conducted the in-depth interview sessions with participants in the study. A third mental health professional doctor experienced in qualitative research provided supervision and expertise for the conduct of the in-depth interview sessions.

The in-depth interviews were conducted face-to-face using a semi-structured interview guide (Table 1).

**Table 1:** Semi-structured interview guide

Semi-structured interview guide
What does the term ‘recovery’ mean to you?
How was your experience with the peer support group (PSG) service?
How does this peer support group (PSG) service affect your recovery?

Other prompts to explore participants’ experiences with the PSG service include:

1. What do you hope to achieve in recovery?
2. How did you know about the service availability?
3. What was your initial perception of the service before joining the session?
4. What was your perception of the service after joining the session? What has changed?
5. What are the advantages/benefits gained from the peer support group sessions?
6. What are the disadvantages/weaknesses of the peer support group sessions?
7. Any suggestions on improvements for the betterment of the peer support group service?

The interview guide consisted of open-ended questions with in-depth probing to allow an exhaustive understanding of the objectives in their own words in order to obtain rich qualitative data. The interviews were conducted in either Malay or English language, according to the participants’ language of preference. Each interview lasted between 30 and 45 min. Data collection was repeated until data saturation was reached. Saturation was established when there were no new themes, ideas or patterns emerged as the interview process progressed. All the in-depth interviews were audio recorded with a digital audio device and then manually transcribed verbatim. For the purpose of reporting, the verbatim in Malay language in this study were translated into English by the first researcher and cross-checked by a second researcher competent in both languages, to ensure the accuracy of translations. A third researcher will be referred to, if any discrepancy arises from the translations.

Following the data collection, data were then analyzed using descriptive thematic analysis [26] which included the following steps: (a) transcribing, reading, and rereading interviews to generate initial ideas; (b) systematic coding of each data set; (c) organizing codes into potential themes; (d) reviewing themes and coded extracts to create a thematic map; (e) refining theme specifics and naming themes to create a coherent story; and (f) writing an analysis report with selected extracts linking back to the research goal [26]. QSR NVivo 12 Computer-Assisted Qualitative Data Analysis Software was used to facilitate data organization. During the data collection and analysis, the researchers’ background, experiences and perspectives may affect the findings. To address reflexivity in this study, the researchers engaged in group consensus to resolve any discrepancies that arise during the process. The descriptive analytic report was developed based on the themes in connection to the objectives of the study. Regular meetings among researchers were done to discuss the themes and subthemes development, and to settle any disputes throughout the analysis process through group consensus.

This study was approved by the Medical Research Committee of National University Malaysia Medical Centre (UKM PP/111/8/JEP-2022-278), and it abides by the regulations of the 1964 Declaration of Helsinki and its subsequent amendments. We have adopted COREQ Guidelines [27] in reporting the research data.

## Results

### Socio-demographic data of participants

There were 11 participants in this study. They were aged between 27 and 56 years, all of which were female and of Malay ethnicity. Most participants were single (*n* = 7), and the rest were married (*n* = 4). Five were employed, while six were unemployed. The primary psychiatric diagnoses for each participant varied (Table 2 on socio-demographic data).

**Table 2:** Socio-demographic data

Participant (*n* = 11)	Gender	Age (year)	Race	Marital status	Psychiatric Diagnosis	Employment status
Par A	Female	54	Malay	Single	MDD	UE
Par B	Female	42	Malay	Single	BMD	E
Par C	Female	36	Malay	Single	Schizophrenia	E
Par D	Female	27	Malay	Single	MDD	E
Par E	Female	51	Malay	Married	BMD	E
Par F	Female	34	Malay	Married	PTSD, BPD	UE
Par G	Female	40	Malay	Married	BMD, BPD	UE
Par H	Female	42	Malay	Single	BMD	UE
Par I	Female	41	Malay	Married	BMD	UE
Par J	Female	56	Malay	Single	Schizophrenia	UE
Par K	Female	36	Malay	Single	BMD	E

Par, participant; MDD, Major depressive disorder; BMD, Bipolar mood disorder; PTSD, Post-traumatic stress disorder; BPD, Borderline personality disorder; UE, unemployed; E, employed

### Perspectives on the meaning of recovery

There are three main themes from the results of the study. Table 3 illustrates the themes and subthemes of the participants’ perspectives on the meaning of recovery.

**Table 3:** Participants’ perspectives on the meaning of recovery

Themes	Subthemes
1. Gaining self-reliance and social inclusion	• Improved social relationships and connectedness
	• Gaining freedom and autonomy
	• Restoring functional independence and daily competence
2. Personal growth and improved life circumstances in recovery	• Career advancement and improved quality of life
	• Self-transformation and personal development
3. Symptoms improvement	• Cognitive symptoms improvement
	• Physical symptoms improvement
	• Psychological symptoms improvement

### Theme 1: gaining self-reliance and social inclusion

#### 1.1 Improved social relationships and connectedness

Improved social relationships and connectedness were identified as one of the meanings of recovery for the study’s participants. The participants described having negative self-perceptions and social isolation in the early part of their mental illness. Therefore, recovery encompasses the transformation in their self-perception and improvement in communication and connection with others, especially family members.

“Before this, I felt sick, lazy, dumb, and ashamed to communicate.” (Participant J).

“Communication with others. Um, I mean communication with family members, or with people outside, or with people we don’t know.” (Participant E).

#### 1.2 Gaining freedom and autonomy

Participants in the study shared how they developed their sense of freedom and autonomy as they went through their treatment, which provided insight into how each of them perceived recovery. The autonomy was mostly described as the ability for illness self-management by the participants and reflected a desire to be liberated from the restrictions imposed by mental illness, and to enjoy a sense of normalcy and autonomy comparable to that of others.

“I don’t want to be admitted to the ward again, and then I keep telling myself, I’m healed. I’m healed. I don’t want to be admitted to the ward again, like that.” (Participant H).

“For me, I no longer need to take medication, I can control my emotions, I can be like, I’m normal just like everyone else.” (Participant I).

#### 1.3 Restoring functional independence and daily competence

Participants in the study shared their perspectives on their desire for functional independence and daily competence in reflecting the meaning of recovery. The ability to gain back premorbid functions and the ability to carry out work-related tasks were shared as the desired outcome of recovery.

“After recovering, I no longer face difficulties in my work, and I can do things smoothly. Sometimes, I tend to forget easily, and I get tired quickly.” (Participant C).

“When I recover, I’m happy because I get myself back to how I used to be. I can function according to my interests, hobbies, and things that I couldn’t do during my illness.” (Participant H).

### Theme 2: personal growth and improved life circumstances in recovery

#### 2.1 Career advancement and improved quality of life

Participants in the study expressed their aspirations for career advancement and improved quality of life, what they would wish for in recovery. Having a career that is able to provide stability, opportunities for growth and secure livelihood was highlighted as the participants’ aim for recovery. The significance of securing a job was described as fulfilling their basic needs and enhancing their quality of life.

“Actually, if possible, I would prefer a job that involves working in an office, like being an office worker or handling various documents.” (Participant C).

“Then, another thing is, I want to work in any job as long as I can have good food, just a normal life without worrying about money, just like that, not aiming to be rich, just ordinary.” (Participant D).

#### 2.2 Self-transformation and personal development

Participants in the study shared their perspectives on self-transformation and personal development as a form of recovery. Personal growth was described significantly by the participants where recovery does not entail returning to one’s former self but rather evolving into a new person. Through the recovery process during which individuals would develop coping mechanisms and inner strength, participants described as becoming more emotionally resilient.

“But for me, it’s like we who were before, we will never be the same as we were before. Recovery doesn’t mean we will become our old selves. Because we can only evolve into something new. Recovery doesn’t equal to before. Recovery is something new. That’s how I see it, don’t know how to explain, hehe (giggles).” (Participant D).

“I feel like I’m getting better. I feel like I’m becoming someone who doesn’t easily cry, someone strong.” (Participant I).

### Theme 3: symptoms improvement

#### 3.1 Cognitive symptoms improvement

The study participants shared improvement in their cognition when asked what recovery means to them. Memory and attention were described to be the most important cognitive domains that participants sought to improve during recovery.

“For me, recovery means, well, the most important thing is to recover in terms of memory. Because if I don’t remember, I can’t do many things.” (Participant A).

“Clear mind.” […] “Sometimes, when I want to think, it feels a bit difficult because there’s a bit of confusion, like my memory wandering off to another place first. It’s not like it used to be. I used to be normal, able to focus. Now I can’t anymore. I can’t focus to be like how I was before, easy.” (Participant C).

#### 3.2 Physical symptoms improvement

Physical symptom improvement was found to be among the meanings of recovery for the study participants. Participants stressed the importance of stabilizing their comorbid physical illnesses such as seizures that can affect their cognitive function and emotion. Furthermore, participants pointed out the debilitating consequences of treatment side effects such as sedation to their physical health.

“So, I have to control my seizures because every time I have a seizure, my memory drops.” (Participant A).

“If I recover, my emotions will be okay. I will feel more calm and energetic, and I won’t feel useless. Usually, on normal days, I feel useless because of the side effects of the medication, which makes me drowsy. So, when I feel drowsy, I sleep. I sleep, and then I become less attentive to my children. In terms of emotions and my interaction with my children, it becomes somewhat limited.” (Participant F).

#### 3.3 Psychological symptoms improvement

The study participants reported psychological symptoms improvement as an integral part of their recovery. The psychological symptoms described varied from mood to psychosis and managing these symptoms reflected improvement of their condition.

“Okay, the answer to that question is more about the stability of my mood. If my mood is stable, I think I have recovered.” (Participant E).

“So, my hope is, I really hope that I don’t hear voices. Because when I hear voices, it really frightens me and makes me feel uncomfortable when I hear them, as they tell me to harm myself. And I have harmed myself many times as well.” (Participant F).

### Perspectives on how peer support group (PSG) aids recovery

Table 4 illustrates the themes and subthemes of how the PSG aids recovery from the perspectives of the study participants.

**Table 4:** Perspective on how peer support group (PSG) aids recovery

Themes	Subthemes
1.Empowerment and growth through peer support	• Instilling hope and positive outlook in recovery
	• Enhancing self-confidence and self-esteem
	• Learning and acquiring new information
	• Self-discovery and better understanding of self
2. Promoting well-being	• Promoting emotional and psychological well-being
	• Promoting physical well-being
3. Social connection and support	• Empathetic and supportive community
	• Reduction of stigma and social isolation
	• Sense of belonging
	• Validation and normalization of experiences

### Theme 1: empowerment and growth through peer support

#### 1.1 Instilling hope and positive outlook in recovery

During the interviews, participants discussed their experiences and aspirations, highlighting PSG impact on instilling hope and cultivating a positive outlook, thus empowering them to pursue their professional goals and reclaim their identities. In some cases, this could be through the expression of determination to gain certain accreditation or re-entering professional programs to continue progressing in their careers.

“I want to get my license back. My pharmacy license.” (Participant D).

“The burden, the burden is like, each of us has become capable of, even though not everyone, about half of us have become capable of not taking those things as burdens. We see them as challenges or, well, challenges.” (Participant H).

#### 1.2 Enhancing self-confidence and self-esteem

The collective experiences of the individuals involved in the study demonstrated that PSG is crucial in enhancing their self-confidence and self-esteem. Participants shared that the activities in PSG had encouraged them to talk to one another and be comfortable enough to speak up and even to express humorous remarks.

“Before, I had ideas to joke around, but I wasn’t very confident in expressing those jokes. So, they stayed buried in my heart.” […] “Yeah. And now, I can crack jokes.” (Participant C).

“Yeah, like me, lacking confidence to speak, but after joining, it’s okay.” (Participant K).

#### 1.3 Learning and acquiring new information

The study participants highlighted the role of PSG in facilitating the acquisition of knowledge and fostering a continuous learning environment for members. PSG serves as a platform for expanding knowledge, exploring new topics, and gaining valuable insights, contributing to other members’ personal growth and recovery journey. It was pointed out that the inclusion of experts and professionals during the group sessions also fostered a rich learning environment. Another added value of PSG highlighted from the participants was the opportunity to learn practical mental health related skills, strategies, and techniques such as distraction methods which were important in their recovery process.

“One thing is that learning is enjoyable. Whenever there’s an opportunity, it’s always great. Every week, I usually learn about 90% new things, especially during the early stages when the doctor is present. And then there are counselors too.” (Participant D).

“Emm, one thing, the teacher taught us early on, it’s a lot about distraction. Because at that time, I was very fragile, with everyday life matters, like that. I learned a lot about distraction.” (Participant H).

#### 1.4 Self-discovery and better understanding of self

The study participants highlighted the transformative journey experienced by them through their participation in the PSG. Individuals would gain insights into their identities, develop self-acceptance, and undergo a profound process of self-discovery through their participation.

“Besides joining with the same experienced speaker, we can understand ourselves. We can understand, we can accept ourselves.” (Participant A).

“Hmm, I’ve changed a lot. I mean, as I mentioned earlier, I used to think that everyone was wrong when it came to me, everyone seemed to be taking advantage of me. But after everything that has happened now, after experiencing what I’ve experienced, I realize that I should focus more on the lessons learned.” (Participant H).

### Theme 2: promoting well-being

#### 2.1 Promoting emotional and psychological well-being

The study participants also highlighted the impact of the PSG on their emotional and psychological well-being. Participations in group meetings fostered a sense of connection with like-minded individuals and demonstrated the empowerment of each individual’s needs and progress, through the support they have garnered in PSG.

“It helps when we have those meetings. Because I notice, if I don’t go to HUKM to meet my peer friends, my mood will be more unstable, like not meeting people who are similar to us, right?” (Participant E).

“Yes, it helps me. Emotionally, when I feel down, my friends give me encouragement, and then we do activities, meaning those activities make us not have time to think about feeling sad or anything, like that.” (Participant F).

“It’s like I was someone who was unsuccessful and sad, but alhamdulillah, all that is gone now. I can say that out of 100%, about 95% of it is already settled.” (Participant H).

#### 2.2 Promoting physical well-being

PSG also promotes physical well-being of the study participants. These statements from the interview transcripts indicated that the PSG has promoted physical health by fostering social engagement, encouraging individuals to leave their homes and participate in activities, thus promoting healthy lifestyle such as weight management.

“And there are also many enjoyable activities, like the gathering we had recently, where we cooked and ate together. Like a potluck. […] Okay. The most obvious benefit is that I can leave the house and not be alone. It allows me to step out of my comfort zone. You know, many psychiatric patients, like me, if you talk to them, they don’t leave their house or even their room. They can’t just go out easily.” (Participant D).

“I travel by MRT, so these things need to be planned. For example, if I have to go somewhere tomorrow, I need to set it up today, like what time to wake up. Even small things need to be considered, like how to get there. But I enjoy it.” (Participant E).

“Alhamdulillah, that group is really suitable for me because even until now, we still take care of our weight. For example, every two weeks, we weigh ourselves to see if our weight has gone up or down.” (Participant F).

“We go out, leave the house, like that. There are activities.” (Participant K).

### Theme 3: social connection and support

#### 3.1 Empathetic and supportive community

The study participants emphasised the importance of an empathetic and supportive community in their recovery process, which the PSG provided. Having others to understand and listen to their emotions or being bonded in PSG through their emotional struggles helped to gain commonality, empathy, respect and affection within the PSG.

“Nowadays, we don’t expect others to solve our problems. We just want others to understand us, to listen to our feelings.” (Participant C).

“There are friends; we can express our feelings. And then, we know that we’re not the only ones feeling sad, in pain, and so on. And then, we share the spirit.” (Participant J).

#### 3.2 Reduction of stigma and social isolation

In aiding participants’ recovery, PSG also helped in reducing the stigma associated with their mental health conditions and managed social isolation. The absence of stigma was observed within the group by its members and how easy it was to make friends within the PSG. Meanwhile some expressed comparable perspectives, underscoring the comprehension and encouragement received from fellow participants.

“Usually, among ourselves, there is no stigma. […] Having friends. We often hesitate to approach people, but here we immediately make friends.” (Participant C).

“When you meet with friends, they give advice; we support each other, friends understand. Before this, I didn’t meet anyone who understood.” (Participant J).

#### 3.3 Sense of belonging

The participants’ data produced a standout finding of a shared sense of belonging within the PSG. They felt loved, accepted, and supported by their peers, which significantly impacted how they felt overall. This acceptance and appreciation for who they were, have fostered a profound sense of belonging and healed their emotional wounds.

“When they accept me, I feel happy. At least, there are people who listen, like I become their mothers, sisters, like that. I feel that way.” (Participant A).

“I mentioned earlier, it’s the feeling of love. Yeah, because we all felt abandoned for a long time, hehe (giggles). We’ve been abandoned for so long, and with everyone trying to cope with their daily routines, all of that, right? There’s no… people say that feeling of love, it’s like, only we ourselves can understand it. So, with our friends, meeting them every two weeks, sometimes every week, it feels like being healed, like a wound. Because we want to talk about our daily problems, our sad moments, it’s impossible to do it every day. It’s impossible to express it. But when we come to peer support, we can chat for a while, and when we go back, when we get hugs from the teachers, it feels like… melting. It feels like the fatigue just disappears momentarily.” (Participant H).

#### 3.4 Validation and normalization of experiences

The PSG validated and normalised each member’s experiences. They found acceptance and understanding by sharing their struggles and similar experiences, for which PSG provided them with comfort and support in doing so.

“So, in this peer support, I see that when there are many people, some of them share the same experience. So, when they have seen the same experience, people can accept it, and we feel comfortable. That is one good thing about peer support.” (Participant A).

“I feel like when we share with each other, you know, among us patients, they understand.” (Participant B).

## Discussion

This qualitative study sought to investigate the perspectives of people living with mental illness who were enrolled in a peer support group on their concept of recovery and the role of peer support in their recovery journey. Through in-depth interviews and thematic analysis, this study uncovered valuable insights into the experiences and perceptions of people living with mental illness, shedding light on the factors contributing to their recovery process and the impact of having peer support in their lives. The present study also contributes to the current literature [28] that highlights the lack and yet important locally produced literature from a personal recovery approach in Asia. The findings of this study highlighted six key themes; three pertaining to the participants’ perspectives on the meaning of recovery (*1. Gaining self-reliance and social inclusion, 2. Personal growth and improved life circumstances in recovery, and 3. Symptoms improvement*) and another three pertaining to how the PSG aids their recovery (*1. Empowerment and growth through peer support, 2. Promoting well-being, 3. Social connection and support*).

The first research question focused on understanding the participants’ perspectives regarding the concept of recovery. The key theme identified was the importance of *gaining self-reliance and social inclusion*, which encompassed sub-themes like *improved social relationships and connectedness*, *gaining freedom and autonomy*, and *restoring functional independence and daily competence*. This finding demonstrated that the PSG provided a supportive environment for which participants could develop communication skills, build self-confidence, and overcome barriers to engagement with others, resulting in a greater sense of belonging, reduced social isolation, and enhanced social connections. Such findings were consistent with previous studies [29, 30], indicating that functional recovery enhances individuals’ sense of self-reliance and fosters social inclusion by enabling them to participate more fully in their communities.

The second theme on *personal growth and improved life circumstances in recovery*, emerged from the sub-themes of *career advancement, improved quality of life*, and *self-transformation and personal development*. Participants expressed aspirations for careers that align with their interests, could provide stability and contribute to their happiness. They also emphasized the importance of economic stability and the ability to meet their basic needs in order to live a satisfying and comfortable life. Recovery, to them, does not involve returning to their former selves but rather transforming into something new. Participants expressed feelings of self-improvement, resilience, and strength, indicating the transformative nature of their journey. This is consistent with the concept of mental health recovery introduced by Deegan, who described it as a unique, non-linear journey shaped by a person’s attitude and approach to the challenges of daily life [31], where it was argued that adults with psychiatric disabilities were not rehabilitated, but rather discovered a new and valued sense of self and purpose, thereby improving their life circumstances.

The third theme on s*ymptoms improvement* focused on the improvements in physical, cognitive, and psychological symptoms experienced by the participants. Effective symptom management is essential in the recovery process, as it allows individuals to function more optimally and engage in meaningful activities. This aligns with Davidson’s concept of “recovery from” serious mental illnesses [32], which involves symptom alleviation and a return to a healthy state post-illness onset. These findings, together with other studies done in Asia [33, 34], highlighted that recovery involves more than just symptom reduction; it is about achieving a fulfilling and a meaningful life despite mental illness challenges. More importantly, this sheds light on the distinct perspectives on the understanding and experience of recovery in the Malaysian cultural contexts, which can guide the focus in implementation of recovery-oriented services, particularly PSG services.

Our second research question explored how study participants perceived the role of PSG in aiding their recovery process, unveiling three main themes. The first theme on *empowerment and growth through peer support* encapsulates the transformative experiences and positive outcomes reported by participants in the study. This finding is consistent with previous studies [35, 36] where PSG offers a valuable resource for individuals with mental health challenges in providing support, empowerment, and opportunities for personal growth. The several sub-themes extended from this theme, shed light on the specific ways in which participants experienced empowerment and growth, by ways of *instilling hope and positive outlook in recovery*, *enhancing self-confidence and self-esteem*, *learning, and acquiring new information*, and *self-discovery and better understanding of self*. Similar to other studies [37, 38], the participants expressed aspirations for personal accomplishments and viewed life’s obstacles as growth opportunities, highlighting the crucial role of PSG in fostering optimism and a positive outlook. PSG also facilitates knowledge acquisition on topics like mindfulness and promoting self-discovery through connections with peers facing similar challenges.

The second theme on how PSG aids recovery speaks of *promoting well-being* and is comprised of two sub-themes: *promoting emotional and psychological well-being* and *promoting physical well-being*. The sub-theme *promoting emotional and psychological well-being* describes how the PSG offers emotional support, encouragement, and a diversion from negative thoughts and emotions. Attending the group meetings and encouragement from peers stabilized the participants’ moods and provided them with a sense of belonging and understanding. The subtheme *promoting physical well-being* emphasizes the group’s impact on the participants’ physical health and well-being. By engaging in enjoyable activities, planning outings, and promoting weight management, members are encouraged to leave their comfort zone at home and socialize outside home, thereby improving their physical health. This demonstrates the group’s beneficial impact on their overall health. This finding aligns with a recent scoping review done [39] studying the impact of PSG being a promising avenue for improving well-being of mental health patients.

The third theme on *social connection and support* consist of the sub-themes: *empathetic and supportive community*, *reduction of stigma and social isolation*, *sense of belonging*, and *validation and normalization of experiences*. Participants highlighted the value of having others who understood and listened to their emotions, even without solving their problems, as it provided a sense of connection and provided emotional relief. Participants also felt less stigmatized about their mental health issues, finding comfort in a non-judgmental and supportive environment provided by their peers. This helped them to combat social isolation that is often associated with mental health issues, supporting existing evidence that the PSG interventions effectively address stigma [40] which has been identified as one of the major barriers to mental health recovery [41]. The group’s acceptance and understanding of them fostered a nurturing and supportive atmosphere. They spoke of feeling ‘like a family’ and appreciated the chance to communicate their struggles, get support, and feel a sense of love and belonging. The group members’ acceptance and sympathy help validate the individual’s experience and create a safe environment to discuss challenges. This finding is consistent with the findings of a recent scoping review, which found that PSG is a potential platform for improving social connections and providing support in mental health settings [39].

While this study provides valuable insights into the perspectives of individuals with mental illness and the role of peer support in their recovery journey, it has some limitations. Firstly, the study was conducted in a specific outpatient psychiatric setting of a tertiary hospital in Kuala Lumpur. Future research could involve multiple mental health service centers at different localities to enhance the representativeness of the results. However, this might be a challenge to implement since PSG services are not routinely available in Malaysian mental health services. Secondly, the participants enrolled in this study are all female and of Malay ethnicity, limiting the generalizability of the findings to the general population. Thirdly, due to the novelty of peer-support services in Malaysia, the resource constraints, and the lack of structure and policies regarding the PSG service, the conventional method of delivering this service via formal PSWs was not possible at the time of writing.

Despite these limitations, this is the first study to investigate the perspective of recovery and how the PSG aids the recovery journey among Malaysian mental health service users living with mental illness. The findings of this study have several implications for mental health practice and policy in Malaysia. Firstly, the findings highlight the importance of a recovery-oriented approach to mental health care that goes beyond symptom reduction to emphasize personal growth, empowerment, and improved quality of life. Mental health services should take a person-centered approach that recognizes each individual’s unique journey to recovery and takes into account their preferences and goals. Secondly, the study highlights the valuable role of PSG in mental health recovery. Integrating peer support programs within outpatient psychiatry clinics can enhance the overall treatment experience for individuals with mental illness. These groups can provide a platform for individuals to share experiences, learn from one another, and build a supportive community that fosters resilience and hope. To foster a supportive and inclusive environment, mental health services in Malaysia should aim to strengthen social support networks and provide accessible PSG services. By developing and promoting a recovery-oriented service like this, Community Psychiatry and Psychosocial Intervention Unit in National University Malaysia Medical Centre has made an important first step in the right direction, and Government-run hospitals that offer psychiatric services can emulate a PSG service as such and make it available to a larger Malaysian population. It would be beneficial for policymakers and key stakeholders in the Malaysian government, particularly those in the Malaysian Ministry of Health, to explore opportunities for integrating this valuable recovery-oriented service into our national mental healthcare system. Finally, regular monitoring of the effectiveness of PSG services and challenges faced by the users and service providers is required to ensure ongoing improvements and quality assurance. Gathering participants feedback and evaluating their progress can help inform program modification that can lead to better outcomes.

## Conclusion

In conclusion, the findings of this study provide valuable insights into the perspectives of mental health users enrolled in a PSG on the concept of recovery, and the role of such service in their recovery journey. The findings demonstrated that the PSG complemented the participants’ perspectives on recovery, reinforcing the notion that a comprehensive and person-centered approach to mental health services is essential for successful and sustained recovery outcomes. The findings add to the growing body of literature on recovery-oriented interventions and have practical implications for Malaysian mental health services, ultimately aiming to improve the well-being and recovery outcomes of individuals with mental health challenges. Future studies can look into the effectiveness of PSG services and the potential generalizability of it to Asian countries and mental health community at large globally.

## Data Availability

The datasets used and/or analysed during the current study available from the corresponding author on reasonable request.

## Abbreviations

PSG: Peer support group
PSW: Peer support worker
HUKM: Hospital Universiti Kebangsaan Malaysia
